# Large extracellular vesicles (microvesicles) in diabetic nephropathy: a systematic review of preclinical studies

**DOI:** 10.3389/fphar.2025.1622280

**Published:** 2025-10-10

**Authors:** Sarah Khalaf Ghanem, Shahenda Salah Abdelsalam, Loulia Bader, Abdelali Agouni

**Affiliations:** Department of Pharmaceutical Sciences, College of Pharmacy, QU Health, Qatar University, Doha, Qatar

**Keywords:** extracellular vesicles (EVs), microvesicles (MVs), microparticles (MPs), diabetes, diabetic nephropathy (DN), systematic review

## Abstract

Diabetic nephropathy (DN) is a significant complication of diabetes and is characterized by progressive kidney damage and dysfunction. Several studies have highlighted the role of a subset of large-sized extracellular vesicles (EVs), commonly known as microvesicles (MVs), as crucial mediators of DN pathophysiology. This systematic review critically evaluates the methodological approaches used to study MVs in experimental models of DN, while also synthesizing the experimental endpoints investigated, to identify consistencies, gaps, and opportunities for standardization. A systematic literature search across PubMed, Embase, and Scopus identified preclinical studies investigating the impact of MVs on renal injury, inflammation, and fibrosis in diabetes. Seven preclinical studies published between 2014 and 2022 met the inclusion criteria. Data extracted: MV origin, isolation/characterization/quantification, models/conditions, dosing/exposure, and endpoints. Seven studies (2014–2022) met criteria. Differential centrifugation predominated for isolation; flow cytometry (FCM) (often Annexin V ± lineage markers), nanoparticle tracking analysis (NTA), and electron microscopy (EM) variably supported identity/size; FCM and NTA were commonly used for enumeration along with protein assays. MV sources included platelets, podocytes, urinary fractions, and MSC-derived vesicles. Across studies, MVs modulated oxidative stress (NOX4/ROS), inflammation (e.g., TNF-α, CXCL7), fibrotic signaling (p38 MAPK/CD36), and cell injury; cargo (e.g., miR-451a) linked to cell-cycle regulators (p15/p19) in early DN. Notable heterogeneity in media depletion, dose reporting, and detection thresholds limited cross-study comparison. We conclude that preclinical evidence supports MVs as early biomarkers and mechanistic drivers in DN, but standardization in isolation, characterization, dosing, and endpoint panels—aligned with MISEV 2023—is needed to enable comparability and translation.

## Graphical Abstract

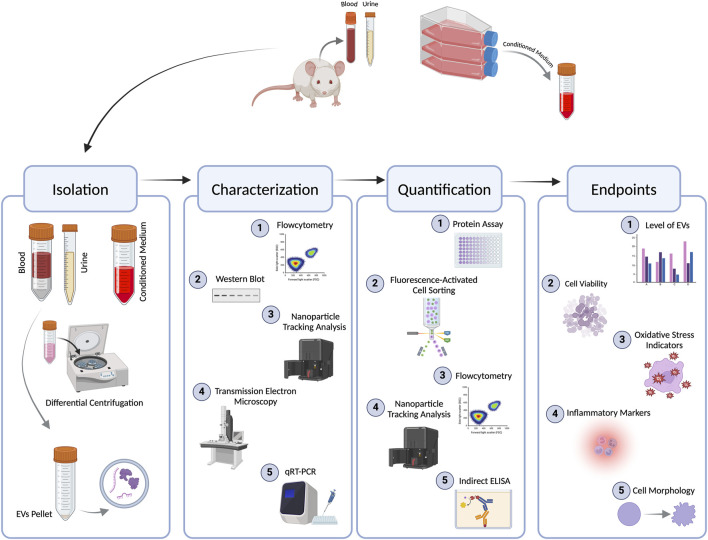

## 1 Introduction

Extracellular vesicles (EVs) are defined as “particles that are released from cells, are delimited by a lipid bilayer, and cannot replicate on their own” (Welsh et al., 2024). EVs were first identified in 1946 in blood plasma by Chargaff and his colleague West; at the time, the authors described them as a ‘particulate fraction’ that sedimented at a speed of 31,000 *g* (Couch et al., 2021). The term ‘extracellular vesicles’ was first used in a scientific publication in 1971 (Witwer and Théry, 2019). Since then, EV-based research has garnered significant attention from scientific researchers in recent years. However, these efforts were hampering the advancement of the field by the presence of significant discrepancies among the different studies in various crucial facets, such as the nomenclature, isolation process, characterization, and enumeration methods of EV subsets. This led to the establishment of the International Society of Extracellular Vesicles (ISEVs) in 2011, with their first guideline published in 2014 (Lötvall et al., 2014), with two consequent amendments published in 2018 and 2023 (Théry et al., 2018; Welsh et al., 2024).

EVs are commonly categorized into three subtypes depending on their size (from smallest to largest): small-sized EVs (sEVs) or exosomes, and large-sized EVs (lEVs), which include microvesicles (MVs), large ectosomes, and apoptotic bodies (Welsh et al., 2024). This nomenclature is of an operational nature as it is based on a physical characteristic such as size. sEVs have a size of <200 nm, while lEVs have a size of >200 nm. Each subtype is released by a different mechanism; sEVs are released through the endosomal pathway, while lEVs are released by direct budding from the plasma membrane, and both can contain versatile cargo from the cell’s cytoplasm (Agouni et al., 2014; El-Gamal et al., 2019; Zaborowski et al., 2015). Despite the recommendations of the ISEVs, research publications continue to use outdated and inaccurate terminology. Methods for isolating and characterizing EVs are crucial for comprehending their biological roles and possible applications. Common techniques for isolating EVs include differential ultracentrifugation (dUC), which separates EVs by density; precipitation methods that use specific reagents to aggregate EVs; and size-exclusion chromatography, which sorts EVs based on size. More recent methods, such as microfluidic devices and immunoaffinity capture, enhance specificity and yield. Once isolated, characterization typically involves techniques like nanoparticle tracking analysis (NTA) to measure size and concentration, flow cytometry (FCM) for surface marker identification, and electron microscopy (EM) for morphological analysis. Additionally, proteomic and lipidomic studies provide important insights into the molecular composition of EVs, revealing potential biomarkers and functional characteristics (Agouni et al., 2014; El-Gamal et al., 2019).

Diabetes mellitus has become a widespread and serious health issue, significantly increasing in prevalence over recent decades. It is associated with various complications, including macrovascular conditions like coronary heart disease and stroke, as well as microvascular conditions such as diabetic nephropathy (DN) (Tomic et al., 2022). DN is one of the most prominent and long-term diabetic complications. It is the primary cause of chronic kidney disease (CKD) and end-stage kidney disease (ESKD) worldwide, accounting for half of all cases. DN is typically defined by the presence of chronic kidney disease in a person with diabetes, characterized by persistently elevated levels of albumin in the urine (albumin-to-creatinine ratio ≥30 mg/g) and/or reduced kidney function (eGFR <60 mL/min/1.73 m^2^) for at least 3 months (Hoogeveen, 2022). Pathologically, the primary characteristics of DN consist of an expansion of the glomerular mesangial matrix, thickening of the basement membrane, glomerular sclerosis, and inflammation and fibrosis in the tubulointerstitial area. While hyperglycemia-induced vascular dysfunction serves as the main trigger for DN, its progression involves various pathological mechanisms, such as inflammation, oxidative stress, autophagy, damage to podocytes, and renal fibrosis (Jin and Zhang, 2024).

EVs have been studied extensively in DN and continue to be a prominent research focus in the field (Tao et al., 2020). Diabetes-induced EVs harness several signaling pathways that contribute to the deterioration of kidney function; for example, encapsulating transforming growth factor beta (TGF-β), mediating its downstream signaling cascade on different kidney cell types (Lu et al., 2020), and mediating endothelial dysfunction through ER-induced cellular stress (Safiedeen et al., 2017; Osman et al., 2020). Research has pinpointed specific EV cargo that indicate renal injury and inflammation, suggesting they could serve as biomarkers for early diagnosis and monitoring disease progression. Some of these discovered biomarkers include microRNA (miR)-21 (Ding et al., 2021), and alpha1-antitrypsin (Ning et al., 2020). Despite the eminent scientific publications on the topic, there remains much to be explored. Overall, the exploration of EVs in DN provides important insights into the disease mechanisms and possible intervention strategies. Several systematic reviews have examined EVs in the context of DN, with an exclusive focus on studies involving human subjects. However, to our knowledge, this is the first systematic review that summarizes and discusses preclinical studies investigating the large-sized EV subset, MVs, in DN.

## 2 Methods

### 2.1 Literature search strategies

We conducted our search using three different databases: PubMed, EMBASE, and Scopus on 8^th^ December 2023. In searching for relevant studies, we utilized Rayyan, an online-based systematic review software, to facilitate the screening and selection process (Ouzzani et al., 2016). This tool allowed for efficient organization and collaboration during the study selection phase. The search strategy used is depicted in Table 1. Two independent reviewers screened the abstracts of retrieved articles and, if necessary, reviewed the full texts. They evaluated the complete articles using specific inclusion and exclusion criteria. Additionally, they examined the references of eligible articles to identify further candidates for inclusion. Any discrepancies were resolved through discussion to reach a consensus.

**TABLE 1:** The search strategy used for the three selected databases.

Database		Search query
PubMed	#1	((microparticles) OR (microvesicles)) AND ((diabetic nephropathy) OR (diabetic kidney disease) OR (DKD))
	#2	(microparticles) AND (diabetes) AND (nephropathy)
	#3	(ectosomes) AND ((diabetic nephropathy) OR (diabetic kidney disease))
	#4	(large extracellular vesicles) AND ((diabetic nephropathy) OR (diabetic kidney disease) OR (DKD))
Scopus	#1	“microparticles” AND “nephropathy” AND “diabetic”
	#2	(TITLE-ABS-KEY (microparticles) OR TITLE-ABS-KEY (microvesicles) OR TITLE-ABS-KEY (large AND extracellular AND vesicles) OR TITLE-ABS-KEY (ectosomes) AND TITLE-ABS-KEY (diabetic) OR TITLE-ABS-KEY (nephropathy) OR TITLE-ABS-KEY (kidney AND disease) OR TITLE-ABS-KEY (diabetes)) AND (LIMIT-TO (EXACTKEYWORD, “*In Vivo* Study”) OR LIMIT-TO (EXACTKEYWORD, “Mice”) OR LIMIT-TO (EXACTKEYWORD, “*In Vitro* Study”) OR LIMIT-TO (EXACTKEYWORD, “Human Cell”) OR LIMIT-TO (EXACTKEYWORD, “Endothelium Cell”) OR LIMIT-TO (EXACTKEYWORD, “Mouse”) OR LIMIT-TO (EXACTKEYWORD, “Animal Model”) OR LIMIT-TO (EXACTKEYWORD, “Animal Experiment”) OR LIMIT-TO (EXACTKEYWORD, “Animals”)) AND (LIMIT-TO (DOCTYPE, “ar”))
Embase	#1	((microparticles) OR (microvesicles)) AND ((diabetic nephropathy) OR (diabetic kidney disease) OR (DKD))
	#2	(ectosomes) AND ((diabetic nephropathy) OR (diabetic kidney disease))
	#3	(large extracellular vesicles) AND ((diabetic nephropathy) OR (diabetic kidney disease) OR (DKD))

### 2.2 Selection criteria

In conducting this systematic review, we aimed to ensure that our review is both rigorous and relevant; hence, specific inclusion criteria were established to match the study objectives. The following criteria were applied: (1) EVs that are termed “microvesicles” or “microparticles” in accordance with the definition of the 2014 minimal information for studies of extracellular vesicles (MISEV) guidelines; for studies using the term “large extracellular vesicles”, the particle size must be within the 200–1,000 nm range; (2) *in vitro*, *ex vivo*, or *in vivo* studies; (3) full-text original research papers. The exclusion criteria were as follows: (1) duplicate articles; (2) other types of EVs, such as exosomes and those exclusively termed apoptotic bodies; (3) types of study: clinical trials, cross-sectional, case-control, and retrospective studies; (4) other diseases; (5) review or commentary; (6) languages other than English.

The grouping of different EVs under one umbrella as lEVs is a recommendation by the latest ISEVs report published in 2023; however, all the included studies were published before the aforementioned time point. Consequently, no critique will be offered regarding the terminology employed by the authors of the studies.

### 2.3 Data extraction

We extracted the following information from each included study: first author, title of the study, publication year, type of study (*in vitro* or *in vivo*), origin of MVs (cell type), methods of isolation, characterization, and quantification of MVs, details of the DN model used, growth medium used for *in vitro* studies, the concentration of MVs used to treat the model of choice, and the treatment time. Concerning the outcomes (i.e., endpoints) of the studies included in this review, we extracted the following: levels of MVs, cell viability, oxidative and nitrosative stress indicators – reactive oxygen species (ROS) levels, and nitric oxide (NO) levels, expression of inflammatory markers, and any renal or cellular morphology-based changes.

### 2.4 Study evaluation

To ensure methodological rigor, the included studies were evaluated against the most recent guidelines of the ISEVs – MISEV2023. These recommendations provide a framework for assessing the quality and reproducibility of EV research, including MVs. Specifically, each study was examined for adherence to the following six criteria. Initially, researchers should deliver quantitative data on both the initial materials and the vesicle yield, encompassing specifics like particle counts, protein levels, and the detection limits of the measurement methods. Next, a thorough evaluation of potential non-vesicular impurities or co-isolated extracellular particles must be involved to confirm that the findings noted are specific to EVs. Third, research should utilize various complementary characterization techniques—like nanoparticle tracking analysis, electron microscopy, or flow cytometry—and supply instrument-specific details, encompassing calibration and detection limits. Fourth, functional assays must include appropriate negative controls, such as EV-depleted supernatants or vesicles disrupted by detergent, and should show both dose–response and time-course effects to verify the specificity of the biological functions observed. Fifth, writers should employ inclusive, practical terminology like “large EV fraction” and steer clear of unsupported assertions regarding vesicle biogenesis unless conclusive proof is presented. Ultimately, for research on EV release and uptake, studies should provide methodological specifics regarding vesicle dosage, incubation duration, labeling techniques, and delivery conditions, especially in *in vivo* environments (Welsh et al., 2024).

### 2.5 Quality appraisal

Given heterogeneity and the absence of a validated tool spanning both *in vitro* and *in vivo* EV studies, we provide a narrative appraisal according to MISEV2023’s checklist.

## 3 Results

### 3.1 Literature search and selection results

**FIGURE 1:**
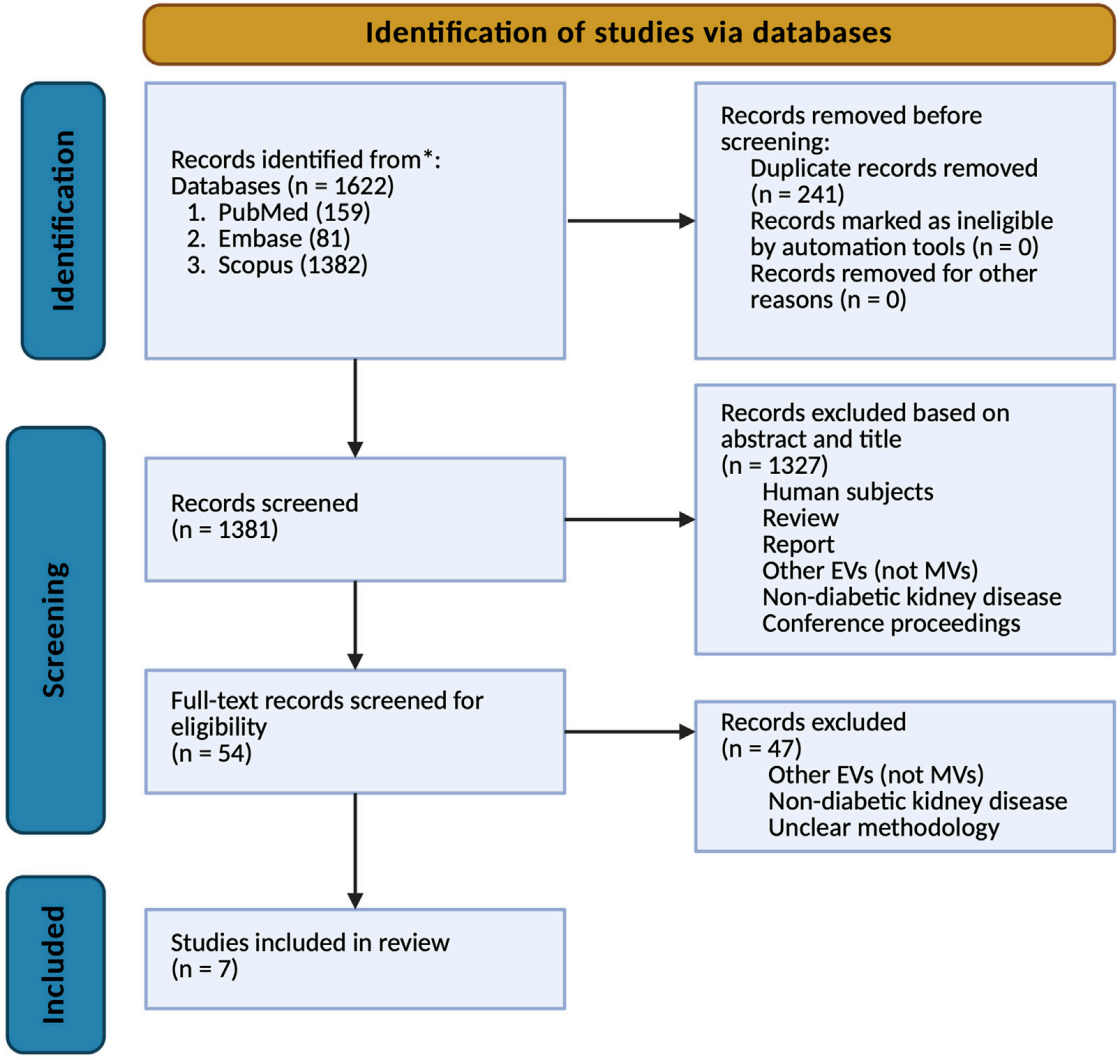
PRISMA flowchart, depicting the literature search process.

The systematic review process was conducted in accordance with the PRISMA (Preferred Reporting Items for Systematic Reviews and Meta-Analyses) guidelines, as illustrated in Figure 1. A comprehensive search of relevant databases was performed to identify preclinical studies meeting predefined inclusion and exclusion criteria. The initial search yielded 1,622 articles, of which 241 were identified as duplicates and subsequently removed. Following the initial screening of titles and abstracts, 1,327 publications were excluded based on various reasons, including human studies, review papers, other EVs studied, non-diabetic kidney disease, and solely diabetes with no nephropathy. The remaining 54 articles underwent full-text evaluation, resulting in the exclusion of an additional 47 studies for similar reasons – other EVs, non-diabetic kidney disease, and unclear methodology. Ultimately, seven studies met all criteria and were included in the final analysis of this systematic review. This rigorous selection process ensured that only the most relevant and methodologically sound studies were incorporated into our analysis, thereby enhancing the reliability and validity of our findings.

### 3.2 Study characteristics

Table 2 summarizes the key characteristics of the eligible studies. The eligible studies were published between 2014 and 2022. Most of the studies were exclusively *in vitro*, except for three studies, which were conducted both *in vivo* and *in vitro*.

**TABLE 2:** Characteristics of the included studies.

No.	Title of the study	Authors	Study type	Year
1	Deposition of platelet-derived microparticles in podocytes contributes to diabetic nephropathy	Huang et al. (2023)	*In vitro*	2022
2	Platelet microparticles mediate the glomerular endothelial injury in early diabetic nephropathy	Zhang et al. (2018)	*In vivo/In vitro*	2018
3	Mesenchymal stem cells–microvesicle-miR-451a ameliorate early diabetic kidney injury by negative regulation of P15 and P19	Zhong et al. (2018)	*In vitro*	2018
4	Podocyte-derived microparticles promote proximal tubule fibrotic signaling via p38 MAPK and CD36	Munkonda et al. (2018)	*In vitro*	2018
5	High glucose provokes microvesicles generation from glomerular podocytes via NOX4/ROS pathway	Li et al. (2019)	*In vitro*	2019
6	Microparticles as Potential Mediators of High Glucose-Induced Renal Cell Injury	Ravindran et al. (2019)	*In vitro*	2019
7	Urinary Podocyte Microparticles Identify Prealbuminuric Diabetic Glomerular Injury	Burger et al. (2014)	*In vitro*/*In vivo*	2014

### 3.3 Quality and methodological assessment of studies

#### 3.3.1 MV origin

In three studies, MVs were isolated from murine models under experimental conditions; two from fresh blood samples (Zhang et al., 2018; Huang et al., 2023) and one from urine obtained from the animals (Burger et al., 2014). Thereby, capturing vesicles present in a systemic context but also introducing heterogeneity due to multiple potential cellular origins. In contrast, the remaining studies relied on conditioned media from defined cell lineages, which provided more controlled vesicle populations and allowed clearer attribution of cellular origin, albeit at the expense of systemic complexity. Notably, Zhang et al. bridged both approaches by culturing platelets isolated from mice and extracting platelet-derived MVs from the supernatant. Thus, while *in vivo* studies provide physiologically relevant vesicle profiles, *in vitro* approaches enable mechanistic dissection under controlled conditions. A summary of the types of MVs isolated in each study and the isolation technique used is shown in Table 3.

**TABLE 3:** Summary of the types of MVs studied, and the isolation, characterization, and quantification techniques used in the seven included studies.

Study authors	Origin/type of MVs	Isolation technique	Characterization technique	Quantification technique	Recipient model treated w/MVs
Huang et al. (2023)	PMPs (mice)	dC	X	Protein assay (BCA)	Immortalized mouse podocyte cell line
Zhang et al. (2018)	PMPs (mice)	dC	FCM (Annexin V, and CD61), EM, and immunoelectron microscopy assay	FCM (beads)	Glomerular endothelial cells
	PMPs (activated platelets/mice)		X	X	N/A
Zhong et al. (2018)	Human umbilical MSCs, and foreskin fibroblasts (CM)	dUC	FCM (beads), zetasizer, and dynamic light scattering particle size analyzer	Protein assay (BCA)	Human renal proximal tubular epithelial cells (HK-2)
Munkonda et al. (2018)	Podocyte MPs (CM)	dC	NTA, WB (synaptopodin, CD63 and TSG101), and qRT-PCR (fibronectin)	X	Human proximal tubule epithelial cells (PTECs)
Li et al. (2019)	MPC-5 (CM)	dC	X	MP-activity ELISA kit	MPC-5 cell line
Ravindran et al. (2019)	NRK-52E (CM)	dC	X	Protein assay (Bradford)	NRK-52E cell line
Burger et al. (2014)	hPOD cell line (CM)	dC	FCM (Annexin V), NTA, and EM	FACS (Annexin V), and NTA	N/A
	Urinary MPs (mice)		FCM (Annexin V, and podocalyxin), and NTA	FACS (Annexin V, and podocalyxin), and NTA	N/A

(X) indicates that the respective technique was not described in the study; CM, conditioned medium; dC, differential centrifugation; dUC, differential ultracentrifugation; FCM, flow cytometry; FACS, fluorescence-activated cell sorting; EM, electron microscopy; NTA, nanoparticle tracking analysis; PMPs, platelet-derived microparticles; WB, Western blot; qRT-PCR, Quantitative real-time reverse-transcription PCR; BCA, bicinchoninic acid assay; N/A, not applicable.

#### 3.3.2 Isolation approaches and implications

Most studies used differential centrifugation (dC) with 10,000–20,000×g pelleting for MVs; Zhong et al. used 100,000×g, a setting that risks co-isolating small EVs and protein complexes, potentially altering downstream readouts (Dilsiz, 2024). Differences in RCF and duration plausibly contribute to variability in yield, purity, and bioactivity across studies.

#### 3.3.3 Characterization of MVs

A multitude of characterization and quantification techniques were used for the isolated MVs in the analyzed studies, as shown in Table 3. Only two studies (Huang et al., 2023; Ravindran et al., 2019) did not employ or describe a characterization technique. The remaining five studies utilized an array of methods to characterize and validate that the isolated fraction consisted of MVs. Three studies used FCM, with minor variations in the fluorochrome-conjugated antibodies or microsphere dimensions utilized; Both Zhang et al. and Burger et al. used Annexin V as a specific marker of MVs; however, Zhang et al. used another more selective marker in combination with Annexin V, CD61 (a tetraspanin). Burger et al used podocalyxin in addition to Annexin V in their animal study to selectively identify MVs that are released from podocytes. Conversely, Zhong et al. employed calibrated polystyrene microspheres (1.0, 0.8, and 0.4 µm) for size-based MV discrimination. Multiple other analytical techniques have been employed across the different studies for EV characterization. NTA, a sophisticated method for high-resolution particle size and concentration measurements, was utilized exclusively by Burger et al. Complementing FCM-based data, Zhong et al. employed dynamic light scattering (DLS) via a Zetasizer for particle size distribution analysis of isolated fractions. EM was utilized by both Zhang et al. and Burger et al. for direct visualization and morphological assessment of EVs. Relative to MISEV 2023 expectations, FCM with Annexin V (± lineage markers like CD61, podocalyxin) and EM/NTA supported MV identity in several studies.

#### 3.3.4 Quantitative assessment of MV yield

For the enumeration of MVs, not all studies succeeded in reporting the method of choice. FCM was utilized by multiple studies, albeit with methodological variations. Zhang et al. employed size-calibrated beads for PMP enumeration, while Burger et al. applied a multi-parameter approach, selecting for Annexin V positivity, size range (0.1–1.0 μm), and podocalyxin expression to identify urinary MVs. Burger et al. further implemented NTA as a complementary quantification method. Protein-based quantification methods were adopted by three studies (Huang et al., 2023; Ravindran et al., 2019; Zhong et al., 2018); both Huang et al. and Zhong et al. utilized the bicinchoninic acid (BCA) assay to measure the total protein content of isolated PMPs, while Ravindran et al. utilized the Bradford assay. Li et al. uniquely employed an Enzyme-Linked Immunosorbent Assay (ELISA) to quantify MVs. Enumeration via bulk protein assays (BCA/Bradford) lacks particle specificity and can misestimate dose. Under-reporting of instrument calibration and detection limits further constrains inter-study comparability.

### 3.4 Experimental conditions – variations in the injury models

#### 3.4.1 *In vivo* – model and regimen conditions

All studies used various models of DN, with some in common (Table 4). Three studies used streptozotocin (STZ)-induced diabetic mice; however, they used different STZ treatment regimens. Huang et al and Zhang et al used a single intraperitoneal injection with a dose of 60 mg/kg depending on the weight of the experimental animal. Burger et al used a different approach where the mice were subjected to five low doses of STZ, intraperitoneally, with a daily dose of 50 mg/kg for five consecutive days. The study by Burger et al. was the only study that used multiple animal models (i.e., four models), where three (STZ-induced, OVE26, and Akita mice) were used to model type 1 diabetes (T1D) and one (db/db mice) was used to mimic type 2 diabetes (T2D).

**TABLE 4:** Summary of the DN model used by the included studies, usage of EV-free growth medium (applicable to studies in which MVs were extracted from cell culture/*in vitro*), the concentration of MVs used to treat the model of choice and treatment duration.

Study authors	Diabetic nephropathy model	Growth medium (EV- depleted ?)	Concentration of MPs used	Treatment time with MVs
Huang et al. (2023)	STZ-induced diabetic mice	N/A	X	X
Zhang et al. (2018)	STZ-induced diabetic mice	N/A	X	X
	30 mmol/L of high glucose, 20 mg/mL of type I collagen, or high glucose plus collagen for 30 min at 37 °C		X	X
Zhong et al. (2018)	High glucose (30 mM) and/or high uric acid (10 mg/dL)	Ultracentrifugation of medium (+FBS) at 100,000 g for 1 h	30 μg	X
Munkonda et al. (2018)	X	Vesicle-free FBS	10 μg/mL	30 min–72 h
Li et al. (2019)	High glucose (30 mM)	X	X	X
Ravindran et al. (2019)	Intermittent high glucose (30 mM/16 h and 5 mM/8 h; for 72 h), and continuous high glucose (30 mM; for 72 h)	Triple filtration of FBS (0.1 µM), and filtration of medium (0.2 µM)	10 μg/mL	24 h
Burger et al. (2014)	High glucose (25 mM), subject to cyclic mechanical stretch, or treated with Ang II or TGF-ß	X	X	X
	T1D model: STZ-induced diabetic mice, OVE26 mice, and Akita mice T2D model: db/db mice	N/A	X	X

(X) indicates that the respective technique was not described in the study; STZ, streptozotocin; N/A, not applicable; FBS, fetal bovine serum; T1D, type 1 diabetes; T2D, type 2 diabetes; ANGII, angiotensin II; TGF-ß, transforming growth factor beta.

#### 3.4.2 *In vitro* – culture conditions

##### 3.4.2.1 High-glucose regimens and co-stimuli

For *in vitro* studies, most studies used high glucose at a concentration of 30 mM; either exposing cells continuously to high glucose (Li et al., 2019), incubating cells intermittently with periods of normal and high glucose (Ravindran et al., 2019), or exposing cells to high glucose in combination with other stimulants such as high uric acid (Zhong et al., 2018) or type I collagen (Zhang et al., 2018). Burger et al. used a high glucose concentration at 25 mM, combined with different kidney injury-causing factors, mechanical stretch, or the addition of inflammatory mediators, angiotensin II, or TGF-β. Munkonda et al. was the only study that failed to state clearly how they modeled DN.

##### 3.4.2.2 EV-free culture conditions: rationale and implementation

The use of EV-depleted culture media is exclusively applicable to *in vitro* experimental models; consequently, its utilization was solely assessed in studies using cell culture systems. In the study by Zhang et al, PMPs isolated from activated platelets *in vitro* were not assessed for this aspect, as platelets do not grow in a traditional culture medium but rather in a buffer, which presumably lacks EV content. Only three studies detailed the use of an EV-free growth medium; Munkonda et al. used a commercially manufactured EV-free Fetal Bovine Serum (FBS). Zhong et al. used a rigorous method of ensuring the depletion of EVs via the ultracentrifugation of the medium at high speed. Ravindran et al. used a filtration technique for both the FBS and the growth medium.

##### 3.4.2.3 MV dosing and exposure scheme

*In vitro* experiments utilizing EVs are highly dependent on the precise quantification and standardization of EV concentrations, as this factor plays a crucial role in determining cellular responses, ensuring the validity of functional assays, and facilitating the replication of findings across diverse experimental conditions. Surprisingly, only three studies described the concentration of MVs used to treat the model of choice, whether a cell line or an animal model. Munkonda et al. and Ravindran et al. used the same concentration (10 μg/mL), while Zhong et al. used 30 µg as an amount with no concentration mentioned. Out of the three studies, only two treatment time settings were specified; Munkonda et al. used a range of 30 min up to 3 days, depending on the endpoint experiment performed, while Ravindran et al. incubated the chosen cell line with the isolated MVs for 24 h.

### 3.5 Endpoints in the context of DN mechanisms

All seven included studies used an array of widely different experimental techniques and methods to investigate the role of MVs in DN (Table 5). Four (Zhang et al., 2018; Huang et al., 2023; Burger et al., 2014; Li et al., 2019) out of the seven studies focused on the change in the level of MVs after the injury model was induced, in comparison to the control. The effect of diabetogenic MVs on the viability and proliferation of cultured cells was explored in three studies (Ravindran et al., 2019; Zhong et al., 2018; Li et al., 2019). Cellular health and kidney function can be effectively assessed through the measurement of oxidative stress markers such as ROS and NO production. These molecular markers serve as crucial indicators, providing significant insights into the development and progression of nephropathy (Jha et al., 2016). Notably, ROS levels were measured only in two studies (Zhang et al., 2018; Li et al., 2019), while NO levels were assessed only in the study by Zhang et al. Various inflammatory markers, e.g., tumor necrosis factor (TNF)-α and C-X-C motif chemokine ligand 7 (CXCL7), were analyzed in all studies except for Burger et al. Morphological and structural alterations in different renal cell types serve as significant indicators of diabetic nephropathy (Cao et al., 2022); such changes were investigated in three studies (Zhang et al., 2018; Huang et al., 2023; Zhong et al., 2018). Huang et al. conducted a comprehensive evaluation, examining both glomerular histopathology and kidney ultrastructure. Zhang et al. concentrated specifically on glomerular histopathology, suggesting a detailed examination of these crucial filtration units. In contrast, Zhong et al. adopted a broader approach by examining kidney sections, with a particular focus on renal tubular epithelial cells and renal interstitial tissue; this method provided a more comprehensive view of kidney pathology.

**TABLE 5:** A summary of the experimental methods and assays employed by the included studies to assess the association of MVs with various DN endpoints.

Study authors	Level of MVs	Cell viability	ROS production	NO production	Inflammatory markers	Morphological changes
Huang et al. (2023)	**+**	**-**	**-**	-	+	+
Zhang et al. (2018)	**+**	**-**	**+**	+	+	+
	**-**		**-**	-	+	-
Zhong et al. (2018)	**-**	**+**	**-**	-	+	+
Munkonda et al. (2018)	**-**	**-**	**-**	-	+	-
Li et al. (2019)	**+**	**+**	**+**	-	+	-
Ravindran et al. (2019)	**-**	**+**	**-**	-	+	-
Burger et al. (2014)	**+**	**-**	**-**	-	-	-

(**+**) indicates utilization of the method by the respective study; (−) indicates that the aforementioned technique was not utilized by the respective study; ROS, reactive oxygen species; NO, nitric oxide.

## 4 Methodological appraisal against MISEV2023

Benchmarked against MISEV2023, only Burger et al. and Zhang et al. delivered strong orthogonal identification (FCM with lineage markers plus EM; Burger et al. also used NTA), whereas several others relied on bulk protein or activity assays for quantification (e.g., BCA/Bradford/ELISA) with limited calibration or detection-limit reporting. Impurity/co-isolate assessment was generally not described. Dose/exposure reporting was clear mainly in Munkonda et al.‘s work (10 μg/mL; 30 min–72 h) and Ravindran et al.‘s (10 μg/mL; 24 h); Zhong et al. reported an amount (30 µg) without concentration, and others lacked dosing detail. EV-depleted media were used or generated in three studies - Zhong et al., Munkonda et al., and Ravindran et al., but not consistently elsewhere. Functional controls (EV-depleted supernatant, detergent-disrupted vesicles, dose–response) were rarely specified. Terminology across studies was pragmatic (MVs/MPs) with no unsupported biogenesis claims. Collectively, these gaps affect interpretation by (i) increasing the risk that observed effects reflect co-isolated material rather than solely MVs; (ii) misclassifying dose when using protein mass or activity units, limiting cross-study comparability and obscuring true dose–response; (iii) allowing serum-derived EV background when EV-depleted media are absent, inflating baseline signals; and (iv) weakening causal inference and specificity in the absence of negative controls and calibrated detection limits. As a result, findings should be weighed as directional rather than definitive, with biomarker claims (e.g., early urinary MVs) and mechanistic links (e.g., ROS, profibrotic signaling) considered plausible but contingent on studies that standardize particle-resolved dosing, impurity controls, and functional assay rigor.

## 5 Models and endpoints—integrated view

Integrating evidence across models is essential to move from signals to mechanisms. *In vivo* readouts indicate when and where renal injury emerges, while *in vitro* systems resolve the cell-type pathways that generate those signals. Accordingly, we align common endpoints (MV abundance, viability, ROS/NO, inflammatory and morphological readouts) and harmonize key methodological descriptors (EV-depleted media, dosing metrics, exposure windows) to map convergent mechanisms and separate true biology from workflow-driven variability.

**FIGURE 2:**
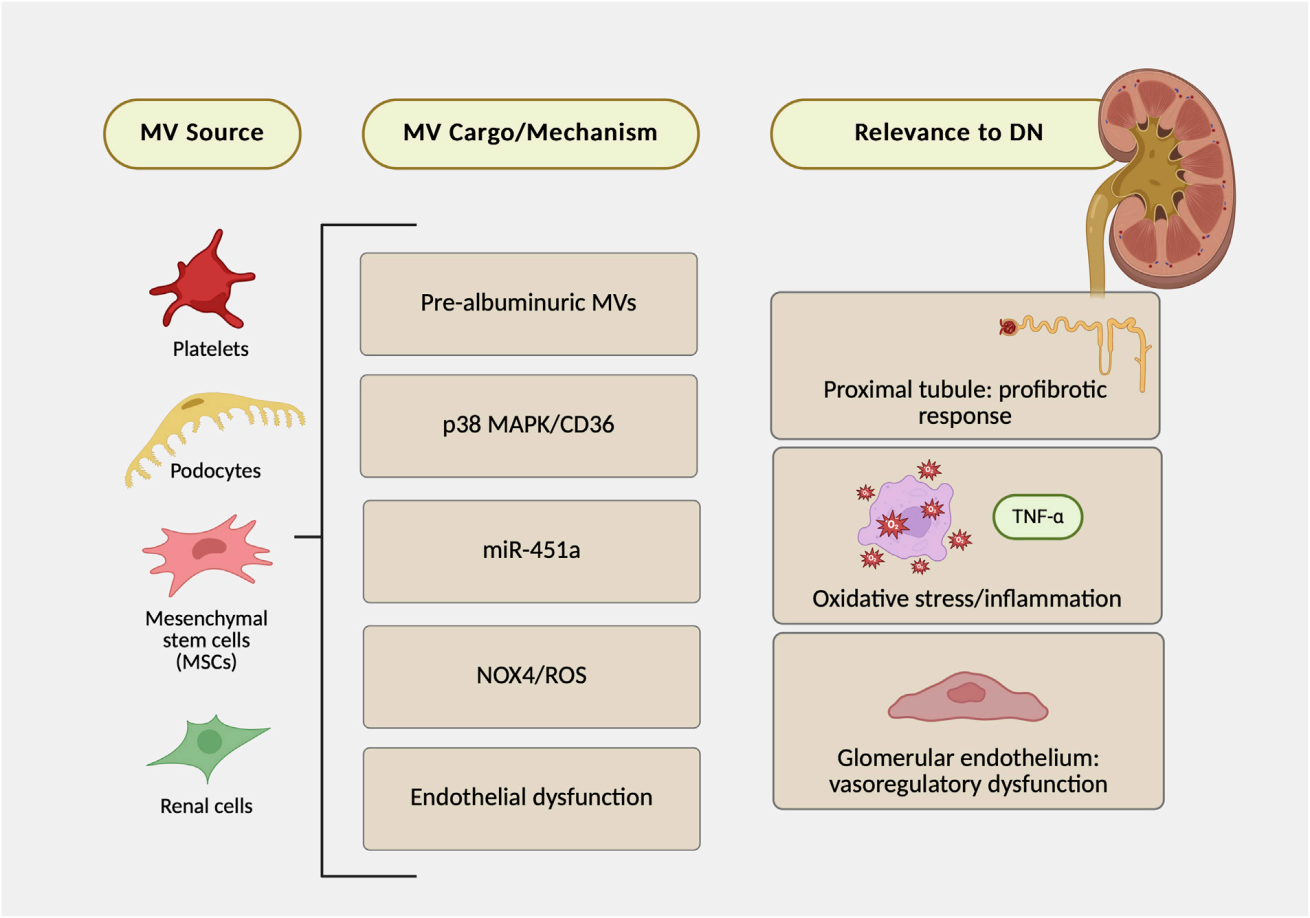
Microvesicles in diabetic nephropathy: evidence map of sources, mechanisms/cargo, and renal outcomes across seven preclinical studies.

In *in vivo* studies (including urinary readouts), MVs consistently point to very early glomerular injury; for example, urinary podocyte MVs rise before albuminuria is detectable, and platelet-derived MVs can be found within glomerular structures. These whole-organ signals pair well with *in vitro* studies that pinpoint cell-type mechanisms: platelet MVs injure glomerular endothelium; podocyte MVs activate p38 MAPK/CD36 in proximal tubules to promote profibrotic signaling; MSC-derived MVs deliver miR-451a that targets p15/p19; and high glucose drives podocyte MV biogenesis through NOX4/ROS. Figure 2 summarizes the key findings from the seven studies and their link to DN. Some of the variability across studies likely comes from methodological differences rather than biology alone—e.g., uneven use of EV-depleted media, different ways of defining dose (protein mass vs. particle counts), and exposure times that range from minutes to days. Tightening these factors (standardized serum depletion, particle-resolved dosing with calibration, and harmonized exposure windows) would make experimental outcomes more comparable across labs and clarify which MV-driven pathways are truly linked to DN progression.

## 6 Discussion

This systematic review identified several key preclinical studies that demonstrate the involvement of MVs in renal injury and inflammation associated with DN. Preclinical systematic reviews and meta-analyses are valuable for uncovering factors that might unintentionally obstruct the translation from bench to bedside, as well as for highlighting variability in the existing literature (Ineichen et al., 2024). In the current review, we have examined and compared various aspects of the selected studies. Firstly, we classified the seven studies according to the experimental model used, *in vitro* or *in vivo*. This led to the identification of the origin/type of MVs explored, with platelet-derived and urinary MVs being the primary sources in *in vivo* studies, while for *in vitro* studies, the only sample type to be collected is the conditioned cell culture medium. Other vital aspects of EV-based studies are the methods of choice for isolation, characterization, and quantification. Besides affecting the yield and purity of EVs isolated, the isolation method can impede their biological function by altering their integrity (De Sousa et al., 2023; Akbar et al., 2022). The results showed that dC is the most common technique used for the isolation of MVs. This observation aligns with existing literature, which indicates that this particular technique is the predominant approach for isolating various subpopulations of EVs across the field (Akbar et al., 2022). The centrifugation speeds used by the selected studies were similar, and this is crucial as the relative centrifugal force (RCF) can affect the purity and yield of MVs; methodologies of included studies stated a range of 10,000 to 20,000 × *g* to efficiently pellet MVs. Furthermore, the duration of centrifugation plays a critical role; longer centrifugation times can enhance separation efficiency but may also risk damaging the MVs if not carefully optimized (Konoshenko et al., 2018). It is important to note that dC is different than dUC; ultracentrifugation is a method that uses extremely high centrifugation speeds, approximately 150,000 × *g*, which is mostly used for small EVs, i.e., exosomes, while dC is a process of consequent rounds of centrifugations with incremental RCF, interrupted by washing steps, to remove cell debris first, and then pellet particles such as MVs (Nigro et al., 2021). MVs, being larger than exosomes, can be isolated using lower centrifugal forces and speeds. Despite the precise description of the centrifugation settings in most of the studies, notably, only a few described the characterization and quantification methods of choice for the isolated particles. Generally, FCM and NTA are some of the most common techniques employed in the included studies and other studies as well for all subsets of EVs (Bağcı et al., 2022). Annexin V, which was used as a marker in most of the included studies, is a widely used marker for characterizing EVs in FCM and Western blotting; it binds to phosphatidylserine, a lipid exposed on EV surfaces during apoptosis. When fluorescently labeled, Annexin V enables quantification and identification of EVs, particularly those from apoptotic cells, providing insights into their origin and function. Other markers were used mostly for identifying a specific type of MVs based on their cell of origin, e.g., podocyte-derived MVs. The EV research community continues to grapple with the challenge of achieving reliable EV quantification, as no standardized method has yet been universally accepted or established. In general, the most widely used methods in recent literature to quantify the number of EVs in a given sample volume and assess their size distribution are NTA and microsphere-based FCM (Hartjes et al., 2019). Another less rigorous method used by research laboratories is colorimetric-based protein quantification assays, which were used by three studies identified in our systematic review. While these assays are widely employed methods for assessing protein concentration, they may exhibit limitations in specificity and sensitivity necessary for precise quantification of EVs. This is primarily due to the complex mixture of proteins and other biomolecules that may be present in the sample, which these assays may not adequately differentiate between and EV-specific proteins. Consequently, this lack of specificity could result in inaccurate estimations of EV levels. Therefore, it is generally viewed as a less reliable method compared to more stringent techniques like NTA or FCM. In terms of EV quantification, one study found that compared to an FCM-based method, there was a significantly lower level of detected proteins, i.e., MVs, in the colorimetric protein assay (Vergauwen et al., 2017).

The seven studies examined employed a diverse array of experimental techniques to investigate various endpoints, highlighting the complex nature of investigating MVs in DN. A significant subset of investigations focused on quantitative alterations in MV levels after renal injury induction, providing foundational data on MV dynamics as potential biomarkers of nephropathic progression. Conversely, a triad of studies explored the *in vitro* effects of diabetogenic MVs on cellular models, elucidating potential mechanisms of MV-mediated cellular dysfunction. These investigations emphasized the production of ROS and NO, key indicators of oxidative and nitrosative stress. However, the limited evaluation of these biomarkers indicates a deficiency in comprehensive oxidative stress profiling. While most included studies assessed inflammatory markers such as TNF-α and CXCL7, their absence in one study suggests methodological inconsistencies across the research landscape. Additionally, the examination of renal cell morphological alterations in select studies underscores the significance of structural parameters in the characterization of DN. The inconsistent findings are largely due to the variations in isolation methods, dose estimation, media preparation, and instrument calibration, which may introduce contaminants or measurement errors. Hence, aligning future studies with MISEV2023 guidelines will help minimize these discrepancies and improve reproducibility.

While MISEV has been essential for rigor, fully implementing the breadth of its recommendations can be rough for many labs and sometimes nearly impossible to execute in full in basic-science settings, given that the 2023 update spans from basic to advanced approaches and even acknowledges that not all conceivable controls can be run simultaneously in any given system. This feasibility gap shows up in field audits: the EV-TRACK analysis of 1,742 EV experiments reported widespread heterogeneity and under-reporting, with <6% of experiments scoring above 50% on its EV-METRIC checklist (Van Deun et al., 2017).

## 7 Conclusion

The collective findings from these studies underscore the critical need for methodological standardization in future MV research within the context of DN. This standardization is essential for enhancing inter-study comparability, improving reproducibility, enabling comprehensive biomarker profiling, integrating morphological and molecular data, ensuring temporal consistency, and establishing uniform quantification methods. The diverse experimental approaches employed reflect both the multifaceted nature of MV research in DN and areas requiring more consistent exploration. Future investigations should address these gaps by establishing consensus on essential biomarkers and cellular parameters, developing standardized protocols for MV isolation and analysis, implementing multi-omics approaches, utilizing advanced imaging techniques consistently, and incorporating *in vivo* models that better recapitulate human DN pathophysiology. By addressing these aspects, the field can progress towards a more unified understanding of MV pathophysiology in DN, potentially uncovering novel diagnostic markers and therapeutic targets. This concerted effort towards standardization and comprehensive analysis will be crucial in advancing our understanding of MVs’ role in DN and translating this knowledge into clinical applications, ultimately improving the management of this complex disease.

## 8 Study limitations

This systematic review has several notable limitations that should be considered. Firstly, the selection criteria may have introduced bias by excluding studies published in languages other than English, which could have led to the omission of relevant findings. Secondly, given the lack of an established quality assessment tool for preclinical non-interventional studies, evaluating the risk of bias was not feasible, which allowed for the possible impact of methodological flaws on the reliability of the conclusions. Moreover, the substantial heterogeneity in the nature and origin of MVs, combined with the wide variability in models used to generate or isolate EVs and the treatment methods involving MVs, constrained our ability to conduct a meta-analysis that could have offered clearer insights. These limitations emphasize the need for caution in interpreting the results and suggest that future research should strive to include a wider range of studies and employ standardized definitions and methodologies for EV isolation and characterization, whether in DN or any other disease model.

## Data Availability

The original contributions presented in the study are included in the article/supplementary material, further inquiries can be directed to the corresponding author.
